# Arbuscular Mycorrhizal Symbiosis as a Promising Resource for Improving Berry Quality in Grapevines Under Changing Environments

**DOI:** 10.3389/fpls.2018.00897

**Published:** 2018-06-29

**Authors:** Nazareth Torres, M. Carmen Antolín, Nieves Goicoechea

**Affiliations:** Unidad Asociada al CSIC (EEAD, Zaragoza, ICVV, Logroño), Grupo de Fisiología del Estrés en Plantas (Departamento de Biología Ambiental), Facultades de Ciencias y Farmacia y Nutrición, Universidad de Navarra, Pamplona, Spain

**Keywords:** berry metabolism, climate change, fruit quality, mycorrhizal fungi, *Vitis vinifera*

## Abstract

Climate change and their resulting impacts are becoming a concern for winegrowers due to the high socioeconomic relevance of the winemaking sector worldwide. In fact, the projected climate change is expected to have detrimental impacts on the yield of grapevines, as well as on the quality and properties of grapes and wine. It is well known that arbuscular mycorrhizal fungi (AMF) can improve the nutritional quality of edible parts of crops and play essential roles in the maintenance of host plant fitness under stressed environments, including grapevines. The future scenarios of climate change may also modify the diversity and the growth of AMF in soils as well as the functionality of the mycorrhizal symbiosis. In this review, we summarize recent research progress on the effects of climate change on grapevine metabolism, paying special attention to the secondary compounds involved in the organoleptic properties of grapes and wines and to the levels of the phytohormones implied in the control of berry development and fruit ripening. In this context, the potential role of AMF for maintaining fruit quality in future climate change scenarios is discussed.

## General Introduction

Grapevine is an important perennial crop worldwide, consumed as fresh or dried (raisins) fruit or produced to make mainly wine, but also grape juice and vinegars. Grapes can also be used for elaborating sweet spreads (marmalade, jelly, butter, and jam). The production of grapevine in Europe achieved of 23.4 million tons (Eurostat Statistics Division, 2016), which represents the 39% of the world production. The moderate consumption of wine (Georgiev et al., 2014; Artero et al., 2015) and the inclusion of grapes and grape products in the diet (Vislocky and Fernandez, 2010) may report beneficial effects for human health because it can positively influence risk factors associated with cardiovascular health, cancer, neurodegenerative disease, and age-related cognitive decline. However, projected climate change is expected to have detrimental impacts on the grapes and wine quality and properties because this crop is highly dependent upon weather conditions during the growing season. The impact of elevated temperatures is one of the environmental factors that most influence both primary and secondary metabolisms and, consequently, the quality of grape berries (Torres et al., 2017).

Mutualistic plant–microbe interactions offer a novel approach to enhance agricultural productivity while reducing environmental costs (Hamilton et al., 2016). Numerous studies have demonstrated that (1) climate change may affect all types of beneficial plant-microorganism interactions and (2) plant-associated microorganisms are an important factor modulating the response of plants to climate change (Compant et al., 2010). Among these beneficial microorganisms, it is worth noting the arbuscular mycorrhizal fungi (AMF), because they can establish mutualistic association with most crops (Smith and Read, 2008) and have an increasingly important role in vineyard production systems (Trouvelot et al., 2015). Although some aspects concerning the effects of global climate change on grapevine cultivation were recently reviewed (Alonso et al., 2016b; Costa et al., 2016; Mosedale et al., 2016; Schultz, 2016; van Leeuwen and Darriet, 2016) so far, the mycorrhizal-mediated responses to grapevine under changing environments have not been updated. In this review, we summarize recent research progress dealing with the effects of climate change on grapevine metabolism and we discuss if the benefits of mycorrhizal symbiosis on berry quality could be maintained under the predicted environmental conditions in future climate change scenarios.

## Climate Change Scenarios for Viticulture

The increasing interest on the effect of climate change on viticulture is not surprising due to the high socioeconomic relevance of the winemaking sector worldwide. Furthermore, climate change and their resulting impacts are becoming a concern for winegrowers (Fraga et al., 2013; Neethling et al., 2017). For most wine-production regions of the world, long-term climate records have shown rising temperatures (Webb et al., 2013; Barnuud et al., 2014; Fraga et al., 2016) together with shifting patterns in rainfall and extreme weather events (Andrade et al., 2012; Intergovernmental Panel on Climate Change, [IPCC], 2014). Warming temperatures have been linked to anthropogenic climate change and are likely to continue. Thus, in most scenarios without additional mitigation efforts, atmospheric concentrations of CO_2_ could reach about 1000 ppm by 2100 and, thus, air temperature is likely to exceed 4°C above pre-industrial levels (Intergovernmental Panel on Climate Change, [IPCC], 2014). This rise of temperature is expected to exert detrimental impacts on grapevine physiology and, consequently, on berry quality (Mosedale et al., 2016), in addition to increase the risk of pests and diseases, especially in areas with warm winters (Caffarra et al., 2012). Heat waves during the growing season enhance the loss of water from the soil thus impairing vine productivity (Schultz, 2016). Moreover, the absence of precipitation, considered as a major limiting factor for plant growth, is frequently accompanied by increased UV-B radiation levels (Bandurska et al., 2013). This new scenario can entail future wine production in areas that are presently too cold for wine cultivation, while the actual grape growing regions may become unsuitable for premium wine production (Hannah et al., 2013; Roy et al., 2017). The establishment of vineyards at higher altitudes (colder areas) and the selection of exposures that lead to a lower interception of solar radiation would be some appropriated options facing these changes (Hannah et al., 2013; Palliotti et al., 2014). On the other hand, in south Mediterranean Europe, climate may limit grapevine yield and berry quality because most of the berry growth and ripening period occurs under conditions of high air temperature and soil water deficit. For this reason, irrigation is expanded fast in this region to mitigate environmental stress and to guarantee stable grape yield and quality (Costa et al., 2016; Resco et al., 2016). Indeed, future strategies to optimize the environmental performance of the viticulture in the Mediterranean region will have to be focused on an adequate selection of rootstocks and phenotypes resistant to drought and heat stress, although currently winegrowers identify them as the last resort strategies (Neethling et al., 2017). Unfortunately, in Mediterranean countries (e.g., Spain) these strategies have been carried out in a non-climate-smart way, this causing that almost a third of the 1 million hectares of grapevines in the Spanish territory will face a different climate to which they were planned (Resco et al., 2016).

## Effects of Changing Environments on the Arbuscular Mycorrhizal Fungi

The plant growth-promoting microorganisms could play vital roles in the maintenance of plant fitness and soil health under stressed environments (Vimal et al., 2017). Climate change related factors may lead to increased C allocation to the root zone, which will potentially alter the composition of root exudates, the C/N ratio or the nutrient availability, with the consequent influence on the composition, abundance and/or activity of plant-associated microbial communities. The data collected by Compant et al. (2010) in their review demonstrated that warming, elevated CO_2_ and drought affect plant-beneficial microorganisms in many ways, the effects being dependent on the climate change factor studied, plant species, ecosystem type, soil type and microbial genotype.

Mohan et al. (2014) summarized the results obtained in research studies dealing with the effect of rising temperatures on mycorrhizal communities. While in 17% of such studies mycorrhizal abundance decreased, in 20% of them no significant change was observed and the 63% of the works concluded that the abundance of mycorrhizas can increase under elevated temperature. In subsequent studies, Augé et al. (2015) pointed out that AMF promotion was 10% higher when air temperatures were kept at or below 27°C than those that exceeded 27°C. Moreover, Wilson et al. (2016) concluded that the direct effect of increasing 3°C the temperature decreases AMF colonization, and this appeared to be regionally consistent across the Mediterranean climate gradient. On the other hand, although the growth of external hyphae and the diversity of AMF species can increase at high temperatures (Hawkes et al., 2008; Zhang et al., 2016), the mycorrhizal activity generally decreases (Mohan et al., 2014). Furthermore, in a warmer world, the presumed enhanced growth of AMF hyphae is unlikely to balance the carbon losses to the atmosphere from the AMF respiration (Hawkes et al., 2008).

Elevated CO_2_ can promote mycorrhizal colonization of plant roots as a consequence of the enhanced carbon allocation to roots (Zhu et al., 2016; Asha et al., 2017), which may result in an increased mineral uptake from soil but not necessarily correlated with nutrient transfer to the host plant (Smith and Read, 2008). Regarding the community composition of AMF, elevated CO_2_ increased the ratio of *Glomeraceae* to *Gigasporaceae* but this effect may be masked by the natural changes through time (Cotton et al., 2015) and also by the dependency of different fungi on water availability and precipitation (Veresoglou et al., 2016). The application of molecular tools revealed that, at present, *Glomeraceae* dominate the composition of the AMF communities in vineyards in Oregon (United States) (Schreiner and Mihara, 2009), Piedmont (Italy) (Balestrini et al., 2010) and Burgundy (France) (Bouffaud et al., 2016), being the AMF diversity relatively low, which contrasts with the high diversity of AMF in the rhizosphere of European wild grapevine *V. vinifera* L. ssp. *sylvestris* (Gmelin) Hegi found by Ocete et al. (2015). Projected droughts within the climate change are expected to cause AMF sporulation and reduction of the AMF activity (Guadarrama et al., 2014). Agricultural practices (high fertilizer inputs, tillage, weed control practices, and pest management practices, among others) may amplify the effect of environmental factors on the AMF communities present in vineyard soils (Trouvelot et al., 2015; Vukicevich et al., 2018). In semi-arid to arid regions, such as Mediterranean areas, soils of vineyards are periodically subjected to tillage or left totally bared in order to keep the soil free of weeds and grassland plant species, which influences on the development and reproduction of AMF. Oehl and Koch (2018) noted that AMF diversity decreased in Central European vineyards subjected to this cultivation management, which can in last instance affect yield and wine quality parameters.

A better understanding of the effect of climatic variability on the synchrony of plants and soil microorganisms which play a key role in the cycle of nutrients and disease cycles is necessary (Pritchard, 2011) and further research on the mechanisms involved in plant-microorganism interactions is required for developing new strategies to manage sustainable agriculture under stressful weather conditions (Vimal et al., 2017).

## Metabolism in Grapevine Berries Under Challenging Environments

Primary and secondary metabolites in grapevine berries are directly involved in the organoleptic properties of grapes and wines (Conde et al., 2007). Climate change is particularly important for berry quality. In fact, drought and light intensity are just some environmental factors that dramatically affect phenolic metabolism and berry chemical composition.

Warming temperatures hasten sugar accumulation and delay color development due to reduction of anthocyanin content (**Table 1**). This decoupling was explained by a relative shift in onset rather than rate of accumulation of these berry components leading to the elaboration of wines with higher alcohol contents (Sadras and Moran, 2012). Moreover, it has been reported that elevated temperatures resulted in higher proportion of acylated anthocyanins (De Rosas et al., 2017). These authors showed that pigment modifications under high temperature are regulated at transcriptional level by MYBA1 transcription factor, and by the UDP glucose: flavonoid-3-*O*-glucosyltransferase and anthocyanin acyltransferase genes. Berry acidity is another important quality trait dependent on the ratio of the concentration between free organic acids (mainly, malic and tartaric acids) and their potassium salt forms. Organic acid metabolism, and especially malic acid concentration, is highly responsive to warm temperatures during fruit ripening. High temperatures are known to induce the degradation of malic acid (Sweetman et al., 2014). Tartaric acid has been thought to be more stable than malic acid; however, several discrepancies were found when the impact of temperature on tartaric acid was studied (**Table 1**). Recent findings of Cholet et al. (2016) showed two groups of expression profiles for the genes involved in the biosynthetic pathway of tartaric acid: those upstream of ascorbic acid, belonging to the Smirnoff-Wheeler pathway and those downstream of ascorbic acid. This study proposed that both groups of genes might be modulated by different environmental factors, which could aid to explain the above mentioned discrepancies on tartaric acid content.

**Table 1:** Berry metabolism response to environmental factors.

Plant material	Region (Country)	Year	Experiment	Environmental factor	Trend	Reference
**Temperature (T)**					
Shiraz, Chardonnay, and Cabernet Sauvignon	Western Australia wine regions	1975–2005	Modeling and projections for 2030, 2050, and 2070	T: Low and high warming condition projected for Australia	CC decreases tartaric acid content and earlier accumulation of sugar is expected. CC reduces anthocyanin accumulation depending on the projected scenario and the cultivar	Barnuud et al., 2014
Sangiovese	n.m.	2010	Potted vines grown in greenhouses	T: +2°C over the average temperature and +7°C over the maximun temperature	T decreased anthocyanin content although no changes in its composition were observed. T did not affect acidity	Movahed et al., 2016
n.m.	n.m.	n.m.	Microvines grown in greenhouses	Day and night HS in different ripening states	Night HS reduced total anthocyanin content. No effect on malate. Night HS up-regulated Pro metabolism related genes	Rienth et al., 2014
Malvec and Bonarda	Mendoza (Argentina)	2014 and 2015	Commercial vineyard	HT: increased mean diurnal temperatures	Day HT reduced total anthocyanins and shifted toward acylated derivatives due to the up-regulation of the acyltransferase gene	De Rosas et al., 2017
Kyoho	n.m.	n.m.	Potted vines in phytotron	T: 25, 27, and 30°C; Shade or sun-exposition	T (27 or 30°C) and shade decreased the anthocyanin content	Shinomiya et al., 2015
**Water deficit (WD)**					
Pinot noir	Geinseihem (Germany)	2009–2011	Field experiment	WS (no rainfall during the growing season) and rootstock	WD increased anthocyanin and other phenolic content in berries but the effect was dependent on the rootstock sensitiveness	Berdeja et al., 2014
Cabernet Sauvignon	Maipo Valley (Chile)	2014	Commercial vineyard	WD (3.6, 1.8, and 0.3 mm day^-1^)	WD increased total phenols and total proanthocyanidins and their polymerization but no differences in total anthocyanins were found	Cáceres-Mella et al., 2017
Chardonnay and Meski	n.m.	n.m.	Potted vines in growth chambers	WD (water privation for 8 days)	WD increased the concentration of Arg, Orn, Glu, Gln, GABA and Pro.	Hatmi et al., 2015
Shiraz and Cabernet Sauvignon	Israel	2011	Field experiment	WD (50% of control)	WD decreased resveratrol and TCA molecules and increased kaempferol and anthocyanins	Hochberg et al., 2015
Shiraz and Cabernet Sauvignon	Negev Desert (Israel)	2011	Commercial vineyard	WD (50% of control)	WD increased amino acid content due to increasing Pro, increased flavonols and anthocyanins and decreased stilbenes and flavanols. WD did not affect Mv derivatives. Between *véraison* and harvest, glucoside derivatives of Dp, Cy, and Pt were increased in Shiraz and only Dp in Cabernet Sauvignon. Coumaroyl forms of anthocyanins were reduced in Shyraz and increased in Cabernet Sauvignon	Hochberg et al., 2015
Tempranillo and Graciano	Navarra (Spain)	2011	Fruit-bearing cuttings grown in greenhouses	WD (50% of control)	WD decreased anthocyanins due to decreasing glucoside derivatives and increasing acetil and coumaroryl derivatives. WD increased flavonols in Tempranillo and decreased flavonols and catechins in Graciano	Niculcea et al., 2015
Shiraz	Montpellier (France)	2004	Experimental vineyard	ED and LD	At maturity, LD increased total anthocyanins, trihydroxylated forms and the acetylated and coumaroylated derivatives. ED increased dihydroxylated forms. Both WD increased non-acylated anthocyanins.	Ollé et al., 2011
Monastrell	Murcia (Spain)	2009–2012	Experimental station	NI, PRI and DI before *véraison*	NI decreased TSS, and increased malic acid, total anthocyanins and the acelylated derivatives. WD enhanced flavonol content until *véraison* and PRI induced amino acid accumulation regardless the amount of water	Romero et al., 2015
Sauvignon vert or Sauvignonasse	Udine (Italy)	2012	Experimental vineyard	NI	WD enhanced phenylpropanoids, monoterpenes, and tocopherols, while carotenoids and flavonoid accumulations were differentially modulated by WD according to the berry developmental stage. WD increased flavan-3-ols and proanthocyanidins before *véraison*, but decreased them after it	Savoi et al., 2016
Merlot	Udine (Italy)	2011 and 2012	Experimental vineyard	WD	WD increased Pro, Leu, Val, and Ile accumulation and decreased the synthesis and concentration of stilbenoids	Savoi et al., 2017
Aglianico	Montegiordano Marina (Italy)	2008	Field experiment	NI	NI increased total anthocyanins and the ratio between acetylated and coumaroylated, flavonols were not affected	Sofo et al., 2012
Tempranillo	Estremoz (Portugal)	2007 and 2008	Field experiment	NI and DI	Sugar accumulation and acidity were not affected. NI increased total phenols but decreased total flavonols, anthocyanins and proanthocyanidins	Zarrouk et al., 2012
**Ligth (L)**					
Riesling	Geisenheim (Germany)	2012	Field experiment	L exposition	L exposition increased flavonol and monoterpene content of berries and their synthases	Friedel et al., 2016
Gamay Fréaux and Gamay	Bordeaux (France)	n.m.	Field experiments	L exposition and exclusion	L exclusion delayed the onset of sugar accumulation by 1 week, decreasing the final concentration of hexose in one cv. L exposition increased anthocyanin concentration after *véraison* whereas L exclusion decreased anthocyanin accumulation (mainly, Dp, Pt, and Mv) in berry skin and flesh	Guan et al., 2016
Cabernet Sauvignon	Negev Desert (Israel)	2014–2015	Field experiment	L exposition (fully, 60% and 30% exposed)	L exposition decreased malic, Asp and fumaric acids while increased tartaric acid in the pulp and triggered the accumulation of Phe, narigenin-chalcone-4-*O*-glucoside, Cy-3-gluc and flavonols and decreased flavan-3-ols, hydroxy-cinnamates and Mv in the skin	Reshef et al., 2017
**UV radiation (UV)**					
Malbec	Mendoza (Argentina)	2008–2009	Commercial vineyard	UV-B	UV-B enhanced anthocyanins, gallic acid, proantocyanins, flavonols and flavanols and decreased TSS	Berli et al., 2011
Grenache and Carignan	Sardinia (Italy)	2009 and 2010	Fields	L exposition and visible R, and visible and UV-A exclusion. Two season, one of them warmer.	UV radiation induced accumulation of anthocyanins with a decrease in trihydroxylated and an increase in dihydroxylated anthocyanins. T caused a decrease in anthocyanin content regardless the L	Fernandes de Oliveira and Nieddu, 2016
Tempranillo	n.m.	n.m.	Fruit bearing cuttings grown under controlled conditions	UV-B: 0, 5.98 and 9.66 kJ m^-2^ d^-1^ applied during two ripening moments	UV-B did not affect sugars and acids. Medium UV-B increased extractable anthocyanins while high UV-B decreased them. UV-B increased flavonols and their mono- and di-substituted derivatives and decreased trisubstituted forms. UV-B did not affect total amino acid concentration although decreased Thr, Met, Ile, Ser and Gly and increased GABA	Martínez-Lüscher et al., 2014
**Temperature (T) and water deficit (WD)**						
Tempranillo	n.m.	2014	Fruit-bearing cuttings grown under controlled conditions.	T: +4°C; WD: ED and LD	T increased glucose and fructose and decreased tartaric acid in berries. Elevated T and LD enhanced amino acid content, mainly Pro, Arg, Thr and Gln. T increased methoxylated anthocyanins and flavonols.	Torres et al., 2017
Tempranillo	Alentejo (Portugal)	2013–2014	Field experiments	SDI and RDI; T: more hours of higher temperature depending on the cluster position	RDI enhanced sugars and decreased acidity, T decreased anthocyanins	Zarrouk et al., 2016
**Temperature (T), and light (L)**					
Gamay	n.m.	n.m.	Cell culture derived from red berry skins	HT: 40°C ; HL: 2500 μmolm^-2^s^-1^	HL decreased anthocyanins although increased Pn and acetilglucoside derivatives and resveratrol. HT increased coumaroyl Pn and epigallocathechin. HT and HL and T increased Trp, Ala and Ser more than 3 fold	Ayenew et al., 2015
*V. lambrusca* (Pione)	Hiroshima (Japan)	n.m.	Research vineyard	L exclusion and T: 35°C	T or dark treatment decreased anthocyanin mainly, Mv and Pn derivatives, and enhanced flavonol content	Azuma et al., 2012
Pione (*Vitis* × *lambruscana*)	n.m.	n.m.	Berries incubated in a multi incubator for 10 days	HT: 35°C, LT:15°C and L (white and UV) or dark	HT or dark decreased anthocyanin accumulation. LT and light induced anthocyanin accumulation	Azuma et al., 2012
Muscat Hamburg	n.m.	2006	Fruit-bearing cuttings grown in greenhouses	HT: 30/25°C (day/night) and HL: (400 μmolm^-2^ s^-1^ PPFD)	T did not affect sugar content, decreased berry TA, malic acid and increased pH. Treatments did not affect amino acid proportion at maturity and total polyphenol content. T decreased anthocyanins and HL increased anthocyanins under elevated T	Carbonell-Bejerano et al., 2013
Pinot noir	Different location in Europe	2013	Field	Latitudinal gradient with changes in T and R	Higher values of solar R decreased phenolic compounds excepting anthocyanins, the ratio between trihydroxylated and dihydroxylated flavonols was strongly correlated with R related parameters. R increased total contents of phenolic groups, mainly flavonols and flavanols	Del-Castillo-Alonso et al., 2016
Carignan and Grenache	Sardinia (Italy)	2009–2011	Field experiment	HT: +1.5°C and 3°C over the average temperature(1971–2000) and attenuation of the PAR and UV radiation	HT decreased anthocyanin content although a positive effect of UV-A on acylation levels was observed increasing the content in Cy and Pn derivatives	Fernandes de Oliveira et al., 2015
**Temperature (T), water deficit (WD) and light (L)**					
Ugni blanc	Cognac region (France)	2011 and 2013	Field	Vintage effect (T, sun radiation and WD: less rainfall)	Warmer, sunnier and dryer vintage increased tartaric and ascorbic acids	Cholet et al., 2016
Touriga nacional and Trincadeira	Pegoes, Setúbal (Portugal)	2007	Fruit-bearing cuttings grown under controlled conditions.	NI (4–5 days without irrigation), HS (42°C, 1 h) and LS (2000 μmol.quanta m^-2^s^-1^, 1 h).	WD, HS and LS increased anthocyanins and carotenoids in leaves from Touriga Nacional	Carvalho et al., 2016
**Ligth (L) and UV radiation (UV)**						
Sauvignon Blanc	Elgin area of South Africa	2012, 2014 and 2015	Commercial vineyard	HL with UV-B attenuation, LL with UV-B attenuation.	UV-B attenuation in HL decreased quercetin-glucoside (responsive of polyphenolic compounds), UV-B exposition enhanced monoterpenes	Joubert et al., 2016
**Water deficit (WD) and UV radiation (UV)**					
Malbec	Mendoza (Argentina)	2011–2013	Commercial vineyard	UV-B, WD and ABA	UV-B increased quercetin and kaempferol	Alonso et al., 2016a
**Climate Change conditions (CC)**					
White and red Tempranillo	Navarra (Spain)	2013	Fruit-bearing cuttings grown in temperature-gradient-greenhouses	CC (T: +4° C over ambient temperature; CO_2_: 700 ppm; CD)	CC decreased malic acid and tartaric acid in white Tempranillo while increased in red. CD and CO_2_ increased sugars in must of both red and white Tempranillo. CC decreased TPI in white, no effect on red Tempranillo. CO_2_ increased total anthocyanins	Kizildeniz et al., 2015
Tempranillo	Navarra (Spain)	2011	Fruit-bearing cuttings grown in greenhouses	CC (CO_2_: 700 ppm; T: 28/18°C; 33–53% RH; WD: 60% of controls) and type of soil	CC increased must pH, and decreased malic and tartaric acid concentrations. CC decreased total anthocyanins and color intensity in the must	Leibar et al., 2017
Tempranillo	Navarra (Spain)	2012	Fruit-bearing cuttings grown in greenhouses	CC (CO_2_: 700 ppm; T: 28/18°C; UV-B: 0; 5,98; 9,66 kJ m^-2^ d^-1^)	UV-B increased flavonols, anthocyanins and UV-absorbing compounds, CC conditions decreased them	Martínez-Lüscher et al., 2015b
Tempranillo	Navarra (Spain)	2008	Fruit-bearing cuttings grown in greenhouses	(T: +4°C over ambient temperature; CO_2_: 700 ppm; WD: 40% of controls)	CC increased TSS, and pH; no effect on the TA or tartaric acid content. WD and CC decreased malic acid. WD decreased anthocyanins, CC mitigated this effect.	Salazar-Parra et al., 2010



*Ala, alanine; Arg, arginine; Asp, aspartic acid; CC, climate change; CD, cyclic drought; CO_2_, Increment of carbon dioxide; Cy, cyanidin; DI, deficit irrigation; Dp, delphidin; ED, early water deficit; GABA, γ-aminobutyric acid; Gln, glutamine; Glu, glutamic acid; Gly, glycine; HL, high light; HS, heat stress; HT, high temperature; Ile, isoleucine; L, light; LD, late water deficit; Leu, leucine; LL, low light; LS, light stress; LT, low temperature; Met, metionine; Mv, malvidin; n.m., not mentioned; NI, non irrigated; Orn, ornithine; Phe, phenylalanine; Pn, peonidin; PRI, partial root irrigation; Pro, proline; Pt, petunidin; R, radiation; Ser, serine; T, Temperature; TA, Total acidity; Thr, threonine; Trp, tryptophane; TSS, Total soluble solids; UV-B, UV-B radiation; Val, valine; WD, Water deficit.*

Several studies have pointed out the role of water deficit on berry quality enhancing total phenolics, particularly, anthocyanins (**Table 1**). Moreover, it has been reported that water deficit changed anthocyanin composition, as well as the composition and the accumulation of flavonols or proanthocyanidins. In general, water deficit enhanced phenylpropanoids, monoterpenes, and tocopherols, while carotenoids and flavonoid accumulations were differentially modulated by water stress according to the berry developmental stage (Savoi et al., 2016), grapevine variety (Niculcea et al., 2015; Kizildeniz et al., 2015) and/or deficit irrigation program applied (**Table 1**). Recent findings showed that the effects of deficit irrigation on berry composition were attenuated at high temperature and that both factors (temperature and deficit irrigation) contributed to modify metabolite profiles of amino acids, anthocyanins and flavonols in Tempranillo variety (Torres et al., 2017). Thus, the combination of elevated temperature and deficit irrigation resulted in high amino acid content mainly due to the accumulation of arginine, proline, threonine, and glutamine. The high arginine and proline contents could be related to a transcriptional regulation of ornithine decarboxylase during water deficit (Berdeja et al., 2015), which could be exacerbated under warm temperatures. Torres et al. (2017) also showed that both temperature and irrigation modified anthocyanin profiles by increasing 3-acetyl-glucosides derivatives due to increased methoxylated forms (petunidin and malvidin). Berry skin flavonols were dominated by myricetin-3-*O*-glucoside but the changes in flavonol profiles were more pronounced at elevated temperatures when plants were subjected to the deficit irrigation. These changes on secondary metabolite profiles could be explained by the regulation at the transcriptional level of phenylpropanoid pathway genes that takes place during water deficit (Castellarin et al., 2007; Deluc et al., 2009) and under elevated temperatures (De Rosas et al., 2017).

Exposure to visible and/or UV radiation is a key factor in the synthesis of phenols and their accumulation in berries. On this subject, several studies have reported increasing trends in flavonols, anthocyanins, flavanols, monoterpenes, and decreases in hydroxy-cinnamates and flavan-3-ols in response to visible, UV-A and/or UV-B radiations (see **Table 1** for further details). However, under climate change scenarios (elevated CO_2_ and temperature) the stimulation of UV-absorbing compound synthesis was reduced (Martínez-Lüscher et al., 2015b). Likewise, the combination of elevated CO_2_, elevated temperature and drought significantly reduced the phenolic content in the same variety, but no effect was observed when the environmental factors were applied individually (Kizildeniz et al., 2015). These results highlight the importance of approach the combined effects of different environmental factors on berry composition.

Grape and wine quality is extremely dependent on the fruit ripening process. Sensory and nutritional characteristics are crucial aspects for wine market, which are developed during berry ripening under a complex hormonal control. Grape berry development involves a complex series of changes, which can be divided into three major phases. Initial berry growth (Phase I) occurs along a sigmoid growth curve due to cell division and subsequent cell expansion. In this phase, the accumulation of organic acids, proanthocyanidins, and hydroxycinnamic acids starts to peak levels. In Phase II (lag phase), cell expansion ceases and sugars begin to accumulate. The beginning of Phase III is marked by the onset on ripening (*véraison*), in which berries undergo a second period of sigmoid growth due to mesocarp cell expansion and accumulate anthocyanin pigments. The accumulation of volatile compounds for aroma and sugars takes place in this phase, as well as the decline in organic acid content and the berry softening.

Several hormones participate in the control of grape berry development and ripening, such as auxin (IAA), ethylene, abscisic acid (ABA), gibberellins (GAs), cytokinins (CKs), and brassinosteroids (BRs) (Böttcher and Davies, 2012). To date, ABA has been the hormone most widely studied in berries in relation to environmental stress factors (**Table 2**). Some studies have reported that berry ABA content diminished under warm temperatures. Likewise, Shinomiya et al. (2015) reported that temperatures exceeding 27°C during the ripening season lead to insufficient berry coloration as a result of low levels of ABA and anthocyanin biosynthetic gene expression levels. Deficit irrigation during growing season also modifies the pattern of hormone accumulation in berries (**Table 2**). In this regard, Niculcea et al. (2013) showed that sustained deficit irrigation (SDI) caused a decrease in ABA and salicylic acid (SA) at *véraison* that affected the amount of anthocyanins at harvest. However, these modifications in berry hormonal patterns also depend on timing of deficit irrigation program applied. Thus, Niculcea et al. (2014) reported that both pre- and post-*véraison* water-deficit modified evolution of ABA, IAA, SA, and JA in berries, which was related to changes in berry size, increases in phenolic substances and accumulation of amines in Tempranillo and Graciano varieties.

**Table 2:** Berry hormones in response to environmental factors.

Plant material	Region (Country)	Year	Experiment	Environmental factor	Berry tissue	Trend	Reference
**Water deficit (WD)**					
Baco noir	Ontario, Canada	2006–2007	Field experiment	NI, and different levels of irrigation at different berry phases	Berry skin and pulp	WD increased ABA and ABA-GE. Irrigation enhanced DPA content	Balint and Reynolds, 2013a
Chardonnay	Ontario, Canada	2006–2007	Field experiment	NI, and different levels of irrigation at different berry phases	Berry skin and pulp	WD increased ABA and ABA-GE in both skin and pulp while decreased PA and DPA content in berry pulp	Balint and Reynolds, 2016
Pinot noir	Geinseingeim (Germany)	2009–2010	Field experiments	WS	Whole berry	WS induced genes related with the JA metabolism	Berdeja et al., 2015
Tempranillo	Navarra (Spain)	2010	Fruit-bearing cuttings grown under controlled conditions	SDI	Whole berry	At the pea-size stage, SDI berries had lower IAA and higher JA and SA than non-stressed berries. At *véraison* (onset of ripening), accumulation of ABA was less accentuated in SDI than in control berries	Niculcea et al., 2013
Tempranillo and Graciano	Navarra (Spain)	2011	Fruit-bearing cuttings grown under controlled conditions	ED and LD	Whole berry	ED caused an earlier ABA peak and LD postponed the peak. ED increased JA and SA concentrations in Tempranillo, and decreased IAA and JA and increased SA at pea size in Graciano	Niculcea et al., 2014
Tempranillo	Estremoz (Portugal)	2007 and 2008	Field experiment	NI vs. DI	Berry skin	NI increased ABA content at *véraison* (in 2007). DI increased ABA concentration in both years	Zarrouk et al., 2012
	
**Temperature (T) and water deficit (WD)**						
Tempranillo	Alentejo (Portugal)	2013–2014	Field experiments	SDI and RDI, more hours of higher temperature depending on the cluster position	Berry skin	HT and RDI decreased free ABA content, the combination between both factors decreased ABA-GE and increased PA and DPA (in 2013) at full maturity	Zarrouk et al., 2016
**Light (L) and UV radiation (UV)**							
Gamay Fréaux and Gamay	Bordeaux (France)	n.m.	Field experiments	L exposition and L exclusion	Berry flesh and skin	Light exclusion reduced free ABA, ABA-GE, PA, and DPA	Guan et al., 2016
**Temperature (T) and light (L)**					
*V. lambrusca* (Pione)	Hiroshima (Japan)	n.m.	Research vineyard	L (white and UV) vs. Dark and elevated T	Berry skin	Elevated T decreased ABA content	Azuma et al., 2012
Muscat Hamburg	Navarra (Spain)	2006	Fruit bearing cuttings grown under controlled conditions	HT (35°C) and HL (400 μmol m^-2^ s^-1^)	De-seeded Berries	HT increased ABA content at different days during ripening	Carbonell-Bejerano et al., 2013
Kyoho	n.m.	n.m.	Potted vines in phytotron	T: 25, 27, and 30°C; and shade or sun-exposed	Berry skin	HT and sun exposure decreased ABA content	Shinomiya et al., 2015



*ABA, abscisic acid; ABA-GE, ABA-glucosylester; CK, cytokinin; DI, deficit irrigation; DPA, dihydrophaseic acid; ED, early water deficit; ET, evapotranspiration; HS, heat stress; IAA, indol-3-acetic acid; JA, Jasmonic acid; L, light; LD, late water deficit; LS, light stress; LT, low temperature; n.m., not mentioned; NI, non irrigated; PRI, partial root irrigation; RDI, regulated deficit irrigation; SA, salicylic acid; SDI, sustained deficit irrigation; T, Temperature; UV, UV radiation; WD, water deficit; WS, water stress.*

## Impact of AMF on the Quality of Crops and Fruits Undergoing Changing Environments

The application of mycorrhizal inocula has emerged as a reliable technique to enhance the agricultural productivity and nutritional value of edible vegetables whereas reducing environmental costs (Berruti et al., 2016; Goicoechea and Antolín, 2017). This is the case of strawberry fruits, whose levels of phenolic compounds and minerals increased when plants were inoculated with the arbuscular mycorrhizal fungus *Glomus intraradices*. Some color parameters of strawberry fruits were also affected by AMF (Castellanos-Morales et al., 2010). Similarly, Hart et al. (2015) found that mycorrhizal inoculation enhanced the concentrations of several minerals (N, P, Cu), carotenoids, and some flavor compounds, as well as the antioxidant capacity in tomato fruits. This beneficial effect of AMF on the quality of tomatoes was corroborated by Bona et al. (2017) in a field study performed in a real industrial tomato farm. In another study carried out under nature conditions, Zeng et al. (2014) found that *Glomus versiforme* improved the quality of citrus fruits by increasing the ratio of sugar to acid, and the amounts of vitamin C, flavonoids and minerals. Another woody plant beneficed by mycorrhization in field is *Libidibia ferrea*, a tree with medicinal properties whose bark accumulated higher amount of flavonoids and tannins when associated with AMF (dos Santos et al., 2017). Mycorrhizal fungi also improved the antioxidant potential of leaves from sweet basil, an aromatic plant widely used for medicinal and cooking purposes (Hristozkova et al., 2017). In lettuce, mycorrhizal symbiosis induced the accumulation of carotenoids, total soluble phenolics, anthocyanins, chlorophylls, tocopherol and some mineral nutrients in leaves (Baslam et al., 2011, 2013a), which makes the application of AMF a feasible tool for improving the nutritional quality of this horticultural crop. This improvement of the quality in lettuces associated with AMF was significant enough to allow extending cultivation of this crop to seasons in which non-mycorrhizal lettuces suffer relevant decreases in their levels of proteins, carotenoids and flavonoids (Baslam et al., 2013b).

However, different factors involved in the expected climate change can modulate or even change the effects of AMF on the metabolism and physiology of their host plants. In fact, drought, salinity, global warming and rising CO_2_ in the atmosphere affect plant growth and yield and constitute a threat to sustainable agriculture and global food security. In studies focused on the role of mycorrhizal symbiosis when plants are undergoing salt stress, some authors have found that AMF increase the plant salt-tolerance and improve fruit yield and quality. This is the case of cucumber cultivated under saline conditions: fruits produced by mycorrhizal plants had higher amounts of soluble proteins, sugars and vitamin C and lower levels of nitrate than those from non-mycorrhizal plants (Han et al., 2012). Likewise, Huang et al. (2013) measured higher contents of N, P, K, and Ca in tomato fruits of hybrid cultivars associated with AMF than in those of non-inoculated ones. Beneficial effects of mycorrhizal symbiosis on yield and fruit quality of crops under salty conditions, however, can vary depending on plant cultivars and fungal strains, as demonstrated by results of Huang et al. (2013) in tomato and those obtained by Sinclair et al. (2014) working with strawberry. Water deficit is one of the most important factors affecting crop survival, growth, and productivity. Most times the beneficial effect of AMF on the host plant development is more evident when water supply is restricted than under plentiful water availability. For example, the beneficial effect of AMF on the growth and quality of chile ancho pepper fruits was especially clear when plants were undergoing drought conditions: fruits of mycorrhizal plants subjected to water deficit showed similar color intensity and chlorophyll content and higher amount of carotenoids than those of non-mycorrhizal plants cultivated at optimal irrigation regime (Mena-Violante et al., 2006). In lettuce, while a moderate water deficit prolonged in time reinforced the capacity of AMF for increasing the levels of antioxidant compounds in leaves (Baslam and Goicoechea, 2012), elevated CO_2_ in the atmosphere impaired this beneficial effect probably due to the use of photoassimilates for enhancing growth of the host plant and spreading mycorrhizal colonization in detriment to the secondary metabolism (Baslam et al., 2012). Similarly, Goicoechea et al. (2016) observed a general depletion of contents of micro- and macro-nutrients and gliadins in grains of durum wheat cultivated under elevated CO_2_ in the air, regardless of mycorrhizal inoculation and water regime applied to plants, what contrasted with the higher accumulation of copper, iron, manganese, zinc and gliadins –wheat-seed storage proteins responsible together with glutenins for dough elasticity and extensibility that determine processing qualities in the production of end products- in grains of durum wheat inoculated with AMF and grown under water deficit at ambient CO_2_. Notwithstanding the above, elevated CO_2_ not always nullify the beneficial effect of mycorrhizal colonization on crop quality. For example, in alfalfa, the combination of AMF and elevated atmospheric CO_2_ improved forage quality by increasing the amount of hemicellulose and decreasing that of lignin in leaves (Baslam et al., 2014). Moreover, the positive effect of the synergism between AMF and elevated atmospheric CO_2_ may be reinforced by the simultaneous application of some cultural practices, such as the supply of humic substances to the soil. In this sense, Bettoni et al. (2014) concluded that the triple interaction between humic substances application, mycorrhizal inoculation and elevated CO_2_ enhanced the accumulation of soluble sugars, proteins and proline in leaves of onion seedlings in a greater extent than the application of those factors separately, which increased the quality of onion shoots as source organs for posterior growth and quality of bulbs. These same authors found that the application of humic substances, AMF inoculum and elevated CO_2_ in the air had an additive effect of increasing the content of soluble sugars, proteins, and phenolics in onion bulbs, thus reinforcing their energetic and antioxidant properties. This triple interaction also enhanced the ratio between soluble solids and total titratable acidity, which may favor the perception of sweetness and make onion more pleasant for consumption (Bettoni et al., 2017).

In addition to the induction of plant defenses, many times through the activation of pathways belonging to the antioxidant and secondary metabolism, Thirkell et al. (2017) suggested that one of the strongest benefits of mycorrhizal symbiosis for crop plants is related to the improved soil properties mediated by AMF. It is known that fungal hyphae increase the mineralisation of soil organic matter (SOM) (Paterson et al., 2016). Moreover, mycorrhizal fungi can enhance the fixation of atmospheric CO_2_ by their host plants, and then induce the transport of photoassimilates from the aerial part to the roots by exerting a sink effect. A portion of the carbon present in the fungal biomass will remain in the soil as a part of the SOM after the AMF senescence (Treseder, 2016). These benefits exerted by AMF will become especially relevant in the context of the projected loss of soil organic carbon caused by the global warming, which in last instance will decrease agricultural productivity (Wiesmeier et al., 2016). Erosion and low organic matter stocks are common problems affecting soils of vineyards in Mediterranean areas as a consequence of the concurrence of environmental factors and some management practices such as the abovementioned continuous tillage (García-Díaz et al., 2018).

### Potentiality of AMF to Mitigate the Negative Impacts of Climate Change on Grapevine Berry Quality

Most studies dealing with the role that mycorrhizal association may play in avoiding or reducing the deleterious effects of the weather conditions predicted for the future have been focused on the secondary metabolism and, mainly, on the phenolic compounds. There is, however, scarce information on the influence of mycorrhizal symbiosis on the levels of phytohormones implied in the berry development and ripening, especially when grapevines are undergoing climatic change scenarios.

Several research works have demonstrated that the association of grapevine with AMF favors the synthesis of plant secondary metabolites -resveratrol, flavonols and anthocyanins, among others-, which are determinant not only for increasing plant tolerance to environmental stresses but also for enhancing berry quality (**Table 3**). In a recent study, Torres et al. (2016) reported that inoculation of grapevines with AMF might play a relevant role in a future climate-change scenario to maintain or even improve berry quality by improving parameters related to the phenolic maturity, such as anthocyanin content, and by enhancing antioxidant activity. This study also highlighted the different abilities of distinct clones from the same variety of grapevine –Tempranillo- to respond to elevated temperature and AMF inoculation. These intravarietal Tempranillo differences were also observed when studied the effects of elevated air temperature, water deficit and mycorrhizal inoculation, separately or in combination, on fruit quality (Torres et al., 2018). However, in all Tempranillo clones used in this study the loss of anthocyanins in berries from non-mycorrhizal plants grown under elevated temperature and subjected to water deficit from the *veraison* to the maturity of berries did not occur in the plants inoculated with AMF.

**Table 3:** Benefits of AMF for grapevines.

Plant material	Experiment	Mycorrhizal presence	Other factors	Effects	Reference
**Plant water status and photosynthesis**					
Asgari, Khalili, Keshmeshi and Shahroodi	Potted vines grown in greenhouses	*Glomus mosseae, Glomus fasciculatum, Glomus intraradices* and a mixture of species		AMF inoculation improved or maintain chlorophyll content	Eftekhari et al., 2012b
Crimson	Commercial vineyard	*Glomus iranicum* var. *tenuihypharum* sp. *nova*	Two years monitoring	AMF inoculation improved the photosynthetic performance, plant water status and increased WUE	Nicolás et al., 2015
Cabernet Sauvignon	Commercial vineyard	n.m.	RDI, ED and LD	AMF inoculation enhanced drought tolerance by compensating the reduced root length due to the more severe water deficits	Schreiner et al., 2007
**Plant growth and nutrient uptake**					
P1103 rootstock- *Vitis berlandieri* × *Vitis rupestris*	Seedlings grown in greenhouses	*Dentiscutata heterogama, G. gigantea, Acaulospora morrowiae, Acaulospora colombiana, Rhizophagus clarus, Rhizophagus irregularis*	Soil with high content in Cu	*R. clarus* and *R. irregularis* improved root dry mass although no effect on chlorophylls was observed	Ambrosini et al., 2015
Cabernet Sauvignon	Field experiment	*Glomus intraradices BEG 72*	Infection by *Armillaria mellea*	AMF inoculation increased plant shoot dry weight	Camprubí et al., 2008
Selection Oppenheim 4 (SO4) rootstock	Potted vines grown in greenhouses	*Glomus intraradices*	Infection by *Xiphinema index*	AMF increased shoot and root mass in both infected or not with the nematode	Hao et al., 2012
Razaki	Potted vines grown outdoors	*Glomus mosseae*	Different N fertilizers	AMF increased shoot dry weight and number of leaves	Karagiannidis et al., 2007
Crimson	Commercial vineyard	*Glomus iranicum* var. *tenuihypharum* sp. *nova*	Two years monitoring	AMF inoculation increased yield and improved quality of grapes	Nicolás et al., 2015
Cabernet Sauvignon	Field experiments	*Glomus intraradices*	Two rootstocks and infection by *Armillaria mellea*	AMF increased total biomass	Nogales et al., 2009b
Pinot noir	Potted vines grown in greenhouses	*G. mosseae, G. intraradices* and *S. calospora*		AMF inoculation improved growth, native AMF were not necessary better than non-native ones	Schreiner, 2007
SO4 and R110 rootstocks	Plants in a growth chamber	*Rhizophagus irregularis*	Infection by *Fusarium oxysporum* f. sp. *herbemontis*	AMF inoculation increased growth as a defense mechanism	Vilvert et al., 2017
P1103 rootstock- *Vitis berlandieri* × *Vitis rupestris*	Seedlings grown in greenhouses	*D. heterogama, G. gigantea, A. morrowiae, A. colombiana, R. clarus* and *R. irregularis*	Soil with high content in Cu	*R. clarus* and *R. irregularis* improved P absorption in contaminated soils	Ambrosini et al., 2015
Razaki	Potted vines grown outdoors	*Glomus mosseae*	Different N fertilizers	AMF modified the mineral concentration of leaves (increased P, K, and B and decreased Zn, Mn, Fe and Cu)	Karagiannidis et al., 2007
Crimson	Commercial vineyard	*Glomus iranicum* var. *tenuihypharum* sp. *nova*	Two years monitoring	AMF inoculation promoted the uptake of P, K and Ca and the mobilization of starch reserves for root development	Nicolás et al., 2015
Pinot noir	Potted vines grown in greenhouses	*G. mosseae, G. intraradices* and *S. calospora*		AMF inoculation improved P, K, Ca, Mg, Fe, and B uptake in some soil, and the nutrient content in stems, leaves, petioles and roots	Schreiner, 2007
**Pathogen resistance**					
Cabernet Sauvignon	Field experiment	*Glomus intraradices BEG 72*	Infection by *Armillaria mellea*	AMF inoculation decreased plant mortality	Camprubí et al., 2008
SO4 rootstock	Potted vines grown in greenhouses	*Glomus intraradices*	Infection by *Xiphinema index*	AMF induced protection against the parasitic nematode decreasing its presence in mycorrhizal roots	Hao et al., 2012
Richter 110 rootstock	Potted vines grown in greenhouses and shadowhouses	*Glomus intraradices*	Infection by *Armillaria mellea*	AMF inoculation provided pathogen resistance	Nogales et al., 2009a
SO4 and R110 rootstocks	Plants in a growth chamber	*Rhizophagus irregularis*	Infection by *Fusarium oxysporum* f. sp. *herbemontis*	AMF inoculation provided pathogen resistance by increasing the expression of defense-proteins	Vilvert et al., 2017
**Metabolism and phenolic content**					
Pinot noir, Divico and Chasselas	Potted vines grown in greenhouses	*Rhizophagus irregularis*	Leaf infection by *Plasmopara viticola* or *Botrytis cinerea*	AMF inoculation increased the active forms of resveratrol, viniferins and pterostilbene.	Bruisson et al., 2016
Asgari, Khalili, Keshmeshi, and Shahroodi	Potted vines grown in greenhouses	*Glomus mosseae, Glomus fasciculatum, Glomus intraradices* and a mixture of species.		AMF inoculation enhanced total phenols and quercetin in leaves.	Eftekhari et al., 2012a
Asgari, Khalili, Keshmeshi and Shahroodi	Potted vines grown in greenhouses	*Glomus mosseae, Glomus fasciculatum, Glomus intraradices* and a mixture of species		AMF inoculation improved total sugars and phenol content in leaves	Eftekhari et al., 2012b
Tempranillo	Potted vines grown in greenhouses	*Glomus intraradices*	T: +4°C	AMF inoculation increased leaf total phenols and total antioxidant capacity, especially at elevated temperature	Torres et al., 2015
Tempranillo	Potted vines grown in greenhouses	*Glomus intraradices*	T: +4°C	AMF inoculation increased must phenolic compounds and total antioxidant capacity, under elevated temperature	Torres et al., 2016
Tempranillo	Potted vines grown in greenhouses	*Glomus intraradices*	T: +4°C, ED and LD	AMF inoculation improved the effects of LD irrigation on grape quality under elevated temperature	Torres et al., 2018
**Gene regulation**					
Pinot noir	Potted vines grown in greenhouses	Two different inocula*: (1) Glomus mosseae (2)* 40% crude inoculum of AMF *(Glomus spp., G. mosseae, and G. viscosum)*, and 21.6% bacteria and saprotrophic fungi		Mycorrhizal inoculation upregulated genes related with nutrient transport, TF, cell wall metabolism in relation with the arbuscular colonization, genes involved in the ABA level. Ethylene responsive factor genes were down regulated	Balestrini et al., 2017
Pinot noir, Divico and Chasselas	Potted vines grown in greenhouses	*Rhizophagus irregularis*	Leaf infection by *Plasmopara viticola* or *Botrytis cinerea*	AMF inoculation up-regulated stilbenoid biosynthesis genes related to defence mechanisms in leaves.	Bruisson et al., 2016
Selection Oppenheim 4 (SO4) rootstock	Potted vines grown under controled conditions	*Glomus irregulare* and *Glomus mosseae*	P starvation	AMF colonization increased genes and proteins involved in carbon metabolism due to P deficiency, P remobilisation, stress and defence, development and root architecture.	Cangahuala-Inocente et al., 2011
SO4 rootstock	Potted vines grown in greenhouses	*Glomus intraradices*	Infection by *Xiphinema index*	AMF up-regulated defence-related *Vitis* genes)	Hao et al., 2012



*Ca, calcium; Cu, copper; ED, early water deficit; Fe, iron; LD, late water deficit; N, nitrogen; P, phosphorus; RDI, regulated deficit irrigation; T, temperature; TF, transcription factor; WS, water stress; WUE, water use efficiency.*

Summarizing the findings of diverse studies, the mechanisms that may be implied in the higher concentrations of secondary metabolites in tissues of plants associated with AMF are the improved photosynthesis and mineral nutrition of host plants, the activation of pathways belonging to the secondary metabolism, the production of signaling molecules, hormonal modifications and/or higher expression of genes involved in the secondary metabolism (dos Santos et al., 2017). In the case of grapevines, several authors have concluded that the positive effects due to AMF colonization are mediated by the up-regulation of some genes (**Table 3**). For example, Balestrini et al. (2017) showed that the expression of genes belonging to categories such as nutrient transport, transcription factors, and cell wall-related genes was significantly altered by AMF colonization. Moreover, the presence of AMF in roots of three grapevine varieties stimulated the transcription of the genes that codify the enzymes phenylalanine ammonia-lyase, stilbene synthase, and a resveratrol *O-*methyltransferase, involved in the response of grapevines to the attack of *Plasmopara viticola* and *Botrytis cinerea* (Bruisson et al., 2016).

## Conclusion and Perspectives

In the last decades, a growing concern about the potential consequences of climate change on viticulture and the detrimental impact on grape and wine quality has addressed several researches. Nevertheless, few studies have highlighted the role of AMF symbiosis in this scenario, in spite of the known benefits that mycorrhizas provide to host plants. The general utilization of fertilizers and/or phytohormones may damage or unbalance soil ecosystem of viticultural areas, so that their application needs to be reduced. Thus, AMF has been presented as natural biofertilizers that can be the alternative to chemical fertilization without the concomitant loss of crop quality (Berruti et al., 2016) and there is evidence that co-adaptation of the partners to a new environment may maximize benefits and minimize costs of the symbiosis (Johnson et al., 2013). On the other hand, exogenous phytohormone application (especially, ABA) to the vine has been used as a tool to improve the quality of the grapes (Balint and Reynolds, 2013b; Alonso et al., 2016a). Taking into account that under abiotic stress ABA concentration was enhanced by AMF (Wang et al., 2017), the symbiotic association can offer an alternative to phytohormone supply to improve grape quality. However, there is a need for more studies that deepen into the influence of AMF in the levels of the ABA when grapevines undergo challenging environments. Moreover, the fact that the responses of grapevines to the inoculation with AMF and/or to the environmental conditions may vary according to the plant variety or clone (Torres et al., 2015, 2016, 2018) indicates that it may be profitable to identify the AMF inoculants most suitable for a given cultivar in a given environment.

Winegrowers are aware of reconsidering their viticultural practices in order to better manage climate-related risks and produce quality wines (Neethling et al., 2017). Given the global warming impact on berry quality traits, it is useful to reconsider the potential application of some new or traditional management techniques able to regulate sugar accumulation and/or to delay or balance berry ripening (Palliotti et al., 2014). Most wine-producing regions are subjected to seasonal drought but, based on the global climate models an increase in aridity is predicted in the future. Hence, an optimized irrigation schedule would still be one of the most desirable tools to improve crop productivity and fruit quality (Costa et al., 2016). Under low rainfall conditions, warm temperatures and high light intensity, spontaneous vegetation used as groundcover appears as an effective strategy to revert soil degradation in Mediterranean vineyards (García-Díaz et al., 2018) at the same time that it may benefit taste or quality of wines (Trigo-Córdoba et al., 2015). Although the impact of water deficit on berry ripening and quality has been extensively investigated during the last decades, the suitability of actual irrigation programs should be reviewed in the future climate conditions. Consequently, several researches have begun to address the combined effects of elevated temperature, UV-B radiation or high CO_2_ with water deficit on grapevine quality (Bonada et al., 2015; Kizildeniz et al., 2015; Martínez-Lüscher et al., 2015a; Torres et al., 2016; Zarrouk et al., 2016). However, more research is needed to elucidate the potential effects of AMF symbiosis on the ability of grapevines to cope with water deficit in interaction with other environmental factors as well as to identify the mycorrhizal inoculants most appropriate for a given variety, cultivar or accession cultivated under a real and specific environmental scenario. In addition, the long-term site history and the previous management practices employed must be considered before introducing the AMF inocula in order to obtain benefits and ensure future food security (Thirkell et al., 2017).

The asexual propagation of the grapevine varieties allows the appearance and accumulation of somatic mutations, which are the basis for the clonal selection, which leads to differences in vigor, berry and cluster weight, yield production, resistance to plagues and diseases or oenological potential (Fernandes et al., 2015). One of the adaptive agronomic strategies to use in modern viticulture under the on-going climate change conditions is the selection of the best adapted rootstock and clones. Thus, the clonal selection could be oriented toward late-ripening clones to avoid alterations caused by high temperatures on fruit quality (van Leeuwen and Darriet, 2016), or to obtain clones with better balance between yield, acidity and alcoholic degree (Gonçalves et al., 2016). Recent findings provide evidence for this clonal diversity, which resulted in different abilities to respond to AMF inoculation (Torres et al., 2016). Therefore, the use of AMF for improving the fruit quality of grapevines needs to be included in an integrated management program of clonal selection.

## Author Contributions

NT carried out the bibliographic search and wrote the first draft of the manuscript. MCA and NG analyzed the related papers and supervised the manuscript. All authors read and approved the submitted version.

## Conflict of Interest Statement

The authors declare that the research was conducted in the absence of any commercial or financial relationships that could be construed as a potential conflict of interest. The reviewer EB and handling Editor declared their shared affiliation.
